# Pharmacological Strategies for Mitigating Cytarabine-Induced Multi-Organ Toxicity: A Scoping Review on Mechanisms, Efficacy and Clinical Implications

**DOI:** 10.3390/cancers18132060

**Published:** 2026-06-25

**Authors:** Ioannis Konstantinidis, Sophia Tsokkou, Kali Makedou, Eleni Gavriilaki, Georgios Delis, Theodora Papamitsou

**Affiliations:** 1 Laboratory of Histology-Embryology, Department of Medicine, Faculty of Health Sciences, Aristotle University of Thessaloniki, 54124 Thessaloniki, Greece; stsokkou@auth.gr; 2 Laboratory of Biochemistry, School of Medicine, AHEPA University Hospital, Aristotle University of Thessaloniki, 54124 Thessaloniki, Greece; kmakedou@auth.gr; 3 Haematology Unit, Haemophilia Centre of Northern Greece, 2nd Propedeutic Department of Internal Medicine, Hippokration General Hospital of Thessaloniki, Aristotle University of Thessaloniki, 54124 Thessaloniki, Greece; gavriiel@auth.gr; 4 Laboratory of Pharmacology, Faculty of Health Sciences, School of Veterinary Medicine, Aristotle University of Thessaloniki, 54124 Thessaloniki, Greece; delis@vet.auth.gr

**Keywords:** cytarabine (Ara-C), systemic toxicity, neurotoxicity, intestinal mucositis, ocular toxicity, hepatotoxicity, nephrotoxicity, developmental toxicity, mitigation, pharmacological strategies

## Abstract

Background: Cytarabine (Ara-C) remains the cornerstone of remission-induction and consolidation chemotherapy for acute myeloid leukemia (AML) and related hematological malignancies. Despite more than six decades of clinical use, its multi-organ toxicity continues to be managed almost exclusively through dose attenuation and supportive care, with no approved upstream pharmacological prevention strategy available. Objectives: This scoping review aimed to systematically map the breadth and nature of pharmacological agents tested in vivo for their capacity to mitigate cytarabine-induced multi-organ toxicity, to characterize their mechanisms of action and organ targets, and to identify evidence gaps and agents with translational potential. Methods: The review was designed and reported in accordance with the PRISMA-ScR checklist. A structured electronic search was conducted across PubMed/MEDLINE, Scopus, Cochrane Library and Embase, and Web of Science from database inception to 15 July 2025. Eligible studies were restricted to full-text, peer-reviewed, English-language research involving in vivo mammalian models administered cytarabine as the principal toxin, with at least one pharmacological co-intervention and at least one quantitative or histopathological organ-injury outcome. Results: From 5701 retrieved records, 36 eligible in vivo mammalian studies (spanning 1964–2024) were identified. Included studies addressed neurotoxicity (*n* = 6), gastrointestinal mucositis (*n* = 9), ocular toxicity (*n* = 3), hepatotoxicity (*n* = 3), bone marrow suppression (*n* = 4), chemotherapy-induced alopecia (*n* = 5), and reproductive and developmental toxicity (*n* = 4). Five recurring mechanistic strategies were identified across the heterogeneous agents tested: redox buffering (N-acetylcysteine, α-lipoic acid, rutin, swertiamarin, α-tocopherol), mitochondrial preservation (betanin, thymoquinone, vitamin D, sodium zinc dihydrolipoylhistidinate [DHLHZn]), tissue-microenvironment reprogramming (apraglutide, BADGE, plerixafor, short-chain fatty acids, β-glucan), molecular antagonism (deoxycytidine, dCMP), and immunomodulation (lienal peptide, IL-1β, AHCC). Conclusions: This scoping review provides the first systematic cartography of pharmacological mitigation strategies for cytarabine-induced multi-organ toxicity. Five mechanistic pathways converge across eight organ systems, with apraglutide and N-acetylcysteine representing the most clinically translatable candidates. Plerixafor and PPARγ blockade by BADGE constitute high-priority candidates for bone marrow niche protection, while the deoxycytidine antagonism principle warrants formal pharmacokinetic evaluation. The complete absence of cardiotoxicity mitigation data defines the most critical gap for future research.

## Simple Summary

Cytarabine (Ara-C) is one of the most important chemotherapy drugs used to treat blood cancers such as acute myeloid leukemia. Although it has been used clinically for more than sixty years, it can cause serious side effects affecting the brain, intestines, liver, eyes, bone marrow, hair follicles, and the developing embryo. Currently, doctors manage these side effects mainly by reducing doses or providing supportive care, but no approved treatment directly prevents the cellular damage that drives them. In this review, we systematically searched the scientific literature to map every drug or compound that has been tested in living animal models to reduce cytarabine-related organ damage. From over 5700 initial records, we identified 36 relevant studies published between 1964 and 2024, covering seven classes of protective agents working through five distinct biological pathways. The strongest signals came from antioxidants, from drugs that restore healthy gut lining, and from agents that reprogram the bone marrow microenvironment after chemotherapy. Two agents, N-acetylcysteine and apraglutide, already have supporting data from human studies and are therefore the most promising candidates for clinical trials in patients receiving cytarabine. Our findings highlight where future research should focus to make cytarabine treatment safer.

## 1. Introduction

Cytarabine occupies a peculiar position in modern oncology pharmacotherapy. More than six decades after its introduction, it remains the backbone of remission-induction and consolidation regimens for acute myeloid leukemia (AML) and a major component of treatment for acute lymphoblastic leukemia, high-grade lymphomas and intrathecal prophylaxis against meningeal disease [1,2,3,4,5]. This pyrimidine analog is intracellularly phosphorylated to its active triphosphate (Ara-CTP), which is then incorporated into DNA in place of deoxycytidine, terminates strand elongation, and ultimately drives dividing cells to apoptosis [1,2]. The same biochemistry that explains the drug’s clinical utility also explains its toxicity: any tissue with a high mitotic index, such as bone marrow, intestinal crypts, hair-follicle matrix, germinal epithelium and embryonic mesenchyme, is intrinsically vulnerable, while tissues whose injury depends on oxidative or mitochondrial damage, such as cerebellum, liver and salivary gland, suffer through related pathways [6].

In our previous systematic review [6], we synthesized the structural and functional consequences of Ara-C exposure across organ systems, drawing on 81 preclinical and clinical studies and consolidating the histopathological signature of the drug across tissues [6]. The clinical reality is that mitigation of cytarabine’s multi-organ toxicity still relies on dose attenuation, leucovorin rescue, intensive supportive care and, in the case of high-dose Ara-C cerebellar syndrome, on close neurological monitoring with treatment interruption when ataxia or nystagmus emerge. None of these strategies act upstream of the molecular events that drive organ injury. They manage damage rather than prevent it.

A growing, although fragmented, preclinical literature has, over the last three decades, examined pharmacological co-administration as a means of decoupling Ara-C’s antineoplastic activity from its off-target injury. The candidate agents are remarkably heterogeneous and span small antioxidants (N-acetylcysteine, α-tocopherol, α-lipoic acid, rutin, swertiamarin), mitochondrial stabilizers (betanin, thymoquinone, vitamin D), microenvironmental modulators of the hematopoietic and intestinal niches (BADGE, plerixafor, apraglutide, β-glucan, glutamine, vitamin A, short-chain fatty acids), classical antagonists (deoxycytidine and its monophosphate), immunomodulators (lienal peptide, AHCC, IL-1β), trichoprotective agents (minoxidil, DHLHZn, ImuVert) and even traditional polyherbal formulations such as the Chinese prescription Guiqi Baizhu. The signals from these studies are sometimes spectacular; apraglutide, for example, rescues survival from 0% to 83–100% in lethal Ara-C mucositis [7], and sometimes equivocal, but together they constitute a body of evidence that no single review has yet mapped systematically. We therefore set out to chart the pharmacological agents that have been tested in vivo for their capacity to mitigate Ara-C-induced multi-organ toxicity, to describe their molecular mechanisms, to summarize their reported efficacy across organ systems, and to identify gaps that should reshape the next decade of translational work in this area.

## 2. Materials and Methods

### 2.1. Protocol and Reporting Standards

A scoping review is the appropriate methodology when the question is not ‘how effective is intervention X versus comparator Y’ but rather ‘what is the breadth, depth and architecture of the evidence base on a given topic, and where are the gaps?’ [8,9]. This scoping review was designed and reported in accordance with the PRISMA-ScR (Preferred Reporting Items for Systematic Reviews and Meta-Analyses extension for Scoping Reviews) 22-item checklist (Table S1) [8] and was structured around the five-stage framework articulated by Arksey and O’Malley [10] and refined by Levac et al. [11] and the Joanna Briggs Institute [9]. A formal a priori protocol was developed by the authors and is available from the corresponding author on reasonable request. No PROSPERO registration was required due to the nature of the study as a scoping review.

### 2.2. Identifying Research Questions

The principal review question was formulated using the Population–Concept–Context framework recommended for scoping reviews [9], as detailed in Table 1. Thus, the following review question was developed: “What is the breadth and nature of pharmacological agents that have been tested in vivo for their capacity to mitigate cytarabine-induced multi-organ toxicity and what mechanisms of action, organ targets and quantitative effects have been reported?” Three sub-questions were also defined: (i) which organ systems have been the focus of mitigation studies, and which remain under-represented; (ii) which mechanistic strategies recur across the literature; and (iii) which agents are closest to translational deployment.

### 2.3. Identifying Relevant Studies

A structured electronic search was conducted in PubMed/MEDLINE, Scopus, Cochrane Library, Embase, and Web of Science from database inception to 15 July 2025, restricted only to the English language. The search algorithm combined cytarabine-related descriptors with mitigation-related concepts, following the structure used in our prior systematic review [6] but augmented with protection-oriented terms. The final string, adapted to each database, was: (“Arabinofuranosylcytosine” OR “Arabinosylcytosine” OR “Aracytidine” OR “Cytosine Arabinoside” OR “Cytarabine Hydrochloride” OR “Cytosar” OR “beta-Ara C” OR “Aracytine” OR “Ara-C” OR “Cytonal” OR “Cytarabine”) AND (“toxicity” OR “side effect*” OR “adverse effect*” OR “neurotoxicity” OR “hepatotoxicity” OR “mucositis” OR “alopecia” OR “myelosuppression” OR “ocular toxicity” OR “pulmonary toxicity” OR “teratogen*” OR “reproductive toxicity”) AND (“protect*” OR “mitigat*” OR “ameliorat*” OR “antioxidant” OR “prevent*” OR “rescue” OR “co-administration” OR “co-treatment” OR “attenuat*”). Reference lists of all included studies and of relevant narrative reviews were reviewed for the identification of any additional relevant articles.

### 2.4. Eligibility Criteria

The review was restricted to full-text research studies published in peer-reviewed journals in the English language. Eligible studies had to meet the following criteria: (i) involved an in vivo mammalian model (rodent, lagomorph or larger species), (ii) administered cytarabine as the principal toxin, (iii) tested one or more pharmacological co-interventions intended to mitigate Ara-C toxicity, and (iv) reported at least one quantitative or histopathological outcome measure of organ injury. Both prophylactic (pre-treatment) and concomitant designs were eligible. Records were excluded if they fell into any of the following categories: duplicates, narrative or systematic reviews, meta-analyses, protocols, guidelines, in vitro studies, human clinical trials of any phase, conference abstracts or presentations, preprints, studies considered irrelevant to the topic, articles with unavailable full texts, and publications in languages other than English.

### 2.5. Study Selection

Once automated tools and the research team applied the exclusion criteria, the remaining articles were exported to a reference management software and deduplicated. To maintain rigor and minimize bias, two independent reviewers (I.K. and S.T.) performed a blinded screening of titles and abstracts. Articles that advanced past this initial stage were retrieved in full and assessed for final eligibility. Disagreements between reviewers at any stage of screening were adjudicated by a third independent reviewer (T.P.).

### 2.6. Data Charting

Key data were independently extracted from all included studies by two reviewers (I.K. and S.T.), comprising the first author and year of publication, cytarabine regimen (dose, route, schedule, cumulative exposure), animal model (species, strain, sex, age, weight), target toxicity assessed, stated study objective, pharmacological mitigation strategy tested (agent, dose, route, schedule), co-treatment outcomes, and the digital object identifier (DOI). Ambiguities were resolved by consensus.

### 2.7. Collating, Summarizing, and Reporting Results

Because scoping reviews are not designed to generate pooled effect estimates, no meta-analysis was attempted. Data were synthesized narratively and grouped by target organ system, then re-sorted by biological mechanism (redox buffering, mitochondrial preservation, niche modulation, molecular antagonism, immunomodulation, trichoprotection) in the Discussion.

## 3. Results

### 3.1. Study Identification and Selection

**Figure 1:**
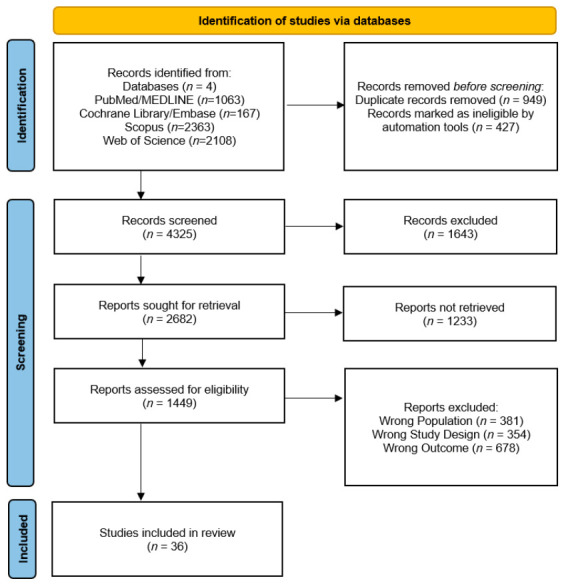
PRISMA flow diagram.

The review selection and exclusion workflow is illustrated using the PRISMA flow diagram (Figure 1). A comprehensive database search yielded 5701 records in total, distributed as follows: PubMed/MEDLINE (*n* = 1063), Scopus (*n* = 2363), Cochrane/Embase (*n* = 167), and Web of Science (*n* = 2108). Following automated screening, 427 records were removed, leaving 5274 for manual evaluation. Of these, 949 duplicates were eliminated, and a further 1643 records were excluded after title and abstract review due to ineligible study designs. An additional 1233 articles could not be retrieved owing to unavailable full texts. The remaining 1449 articles underwent full-text review, from which 36 studies fulfilled all inclusion criteria and were ultimately incorporated into the review.

The 36 included studies span the period 1964 to 2024 and report data from rodent (rat, mouse), lagomorph (rabbit) and germ-free mouse models. Group sizes range from three to thirty-five animals per arm. The geographic distribution of the corresponding authors spans North America, Europe, Asia and South America, but the methodological architecture is overwhelmingly that of a single-laboratory, single-agent, short-duration proof-of-concept study.

### 3.2. Distribution of Studies Across Organ Systems

Mitigation studies were not evenly distributed across the organ systems that suffer Ara-C toxicity. The greatest concentration of evidence was on gastrointestinal mucositis (nine studies), neurotoxicity (six studies), hepatotoxicity (three studies), and chemotherapy-induced alopecia (five studies). The bone-marrow and hematopoietic compartment was addressed by four studies, the eye by three, the reproductive system and developmental toxicity by four, and the lung and salivary gland by one each. No identified study tested a pharmacological strategy to mitigate cytarabine-induced cardiotoxicity, which constitutes the principal gap noted in the present review. Table 2 summarizes all studies included in this review.

### 3.3. Neurotoxicity

Cytarabine neurotoxicity, dominated by cerebellar ataxia at cumulative high doses, was addressed in seven studies that collectively converge on two mechanistic claims. Koros et al., in two complementary investigations in adult Wistar rats in 2007 and 2009, established that 400 mg/kg/day intraperitoneal Ara-C for five days produces ataxic gait, rotarod deficits, Purkinje-cell monolayer disruption, granule-cell loss, and a selective 40% reduction in the high-molecular-weight neurofilament subunit NF-H, while sparing NF-M and NF-L [28,29]. Oral N-acetylcysteine (NAC) at 200 mg/kg/day, started seven days before and continued throughout Ara-C dosing, fully prevented the behavioral and cytoskeletal alterations in both studies. The mechanistic rationale, which is the replenishment of reduced glutathione and direct scavenging of reactive oxygen species generated by Ara-C-injured cerebellar neurons, is biologically coherent, internally consistent, and well-supported by the two-way ANOVA designs that the authors employed.

Salimi et al., in 2023, extended the redox argument to the mitochondria. In adult male Wistar rats exposed to 70 mg/kg/day Ara-C for five days, betanin (25 mg/kg/day), vitamin D (500 IU/kg/day) and thymoquinone (0.5 mg/kg/day) each normalized acetylcholinesterase and butyrylcholinesterase activities, reversed the depletion of reduced glutathione and the rise in malondialdehyde and oxidized glutathione, restored mitochondrial succinate dehydrogenase activity, attenuated mitochondrial swelling, normalized reactive oxygen species generation and prevented collapse of mitochondrial membrane potential [32]. The histopathological recovery, determined by restoration of neuronal cytoplasm, nuclear detail and granule-layer architecture in the midbrain, matched the biochemical recovery.

Guzmán et al. have, over three studies in the young Wistar rat model, mapped a related but distinct pathway. Oleic acid (1500 µL/kg) co-administered with Ara-C (70 mg/kg for 5 days) partially restored regional brain glutathione, Na^+^, K^+^-ATPase activity and dopamine metabolism [26]. Stevia (0.6 g/kg) given 30 min before high-dose intravenous Ara-C (0.6 g/kg × 5 days) attenuated TBARS rises, restored glutathione and modified striatal monoamines in a region-specific fashion [25]. A polyvalent oligoelement supplement (Fe, Zn, Mn, Se, Cr, Cu, Mo, I, F) reduced lipid peroxidation in cortex, striatum and cerebellum and altered Na^+^, K^+^-ATPase activity and regional dopamine after a single low-dose Ara-C exposure [40].

### 3.4. Gastrointestinal Mucositis

Cytarabine mucositis is the organ-system area in which preclinical mitigation evidence is densest, and it is also the area in which the pipeline to the clinic is most clearly visible. Nine included studies tested seven distinct interventions, and they together describe a mucosa under siege from multiple, partially independent insults: direct crypt-cell antimetabolite toxicity, epithelial barrier disruption, M1-polarized macrophage inflammation, microbial dysbiosis and loss of nutrient-driven trophic signaling.

The most interesting result came from Minden et al. in 2024, who tested the long-acting GLP-2 analog apraglutide in mice exposed to cytarabine 30 mg/kg twice daily for five days [7]. The Ara-C-alone group experienced 100% mortality by day three. Apraglutide co-administration at 3.3 mg/kg, started four days before chemotherapy, achieved 83–100% survival, with preservation of villous and crypt architecture, maintained plasma citrulline as a biomarker of enterocyte mass, and normalized the gut microbiota. The dose–response analysis showed near-maximal protection from 1.1 mg/kg, with weaker effects at 0.33 mg/kg and from native human GLP-2.

Li et al. in 2023 proposed a complementary pathophysiological mechanism. Astragaloside IV, the principal bioactive saponin of Astragalus membranaceus, attenuated weight loss and ileal histological damage, restored Occludin/ZO-1 and normalized TNF-α, IL-6 and IL-10 at 10, 20 and 40 mg/kg in C57BL/6 mice exposed to 100 mg/kg/day Ara-C for seven days, with the 40 mg/kg dose producing the most complete protection [18]. The mechanism, dissected by flow cytometry and Western blotting, was a reduction in M1 macrophage polarization in the ileum and downregulation of PI3K/AKT signaling, confirmed in vitro by AKT-siRNA knockdown and molecular docking. Chu et al., in 2023, working in the same C57BL/6 model with the polyherbal Guiqi Baizhu prescription, arrived at a closely overlapping conclusion: suppression of M1 macrophage polarization with reduced M1 markers (CD86+, iNOS+F4/80+) through inhibition of JAK2/STAT1 signaling [24].

Three independent investigations converge on the protective effect of luminal short-chain fatty acids and related substrates. Ramos et al., in 1997, showed in conventional Swiss mice that oral acetate, propionate and butyrate co-administration reduced villous atrophy and necrosis with preserved villus height, but no change in crypt depth and maintained mucosal protein and nucleotide levels on both standard chow and an elemental diet [20]. In a follow-up study in germ-free mice in 1999, the same group confirmed that the protection was independent of endogenous microbial SCFA production and required exogenous delivery [38]. De Souza Silva et al. in 2018 and Porsani et al. in 2017, both showed that oral β-D-glucan and glutamine, administered for 21 days before Ara-C challenge in BALB/c mice, halved leucopenia, reduced comet-assay DNA damage in blood leucocytes by more than 50%, preserved crypt mitotic activity and restored the IL-10/IFN-γ cytokine balance [14,35]. Elli et al., in 2009, added vitamin A (5000 IU/kg orally daily for seven days) to the protective armamentarium, showing preserved jejunal villus length and crypt depth and reduced inflammatory infiltration relative to Ara-C alone [36].

Chen offered the only mitigation study that targets a functional rather than a structural endpoint of intestinal mucositis. In Swiss-Webster mice exposed to Ara-C 50 mg/kg/day for five days, the drug reduced intestinal absorption of D-glucose, 3-O-methyl-glucose and L-tyrosine by 60–74% and disrupted Na^+^ and Cl^−^ transport in Ussing-chamber assays [44]. Co-administration of 2′-deoxycytidine (100 mg/kg) restored 3-O-methyl-glucose transport to control and prevented weight loss, establishing the principle, first explored more than half a century ago, that an exogenous deoxycytidine pool can rescue tissues from Ara-C antimetabolite injury without abolishing the antineoplastic effect.

### 3.5. Ocular Toxicity

Three studies, spanning sixty years, address Ara-C ocular toxicity. Kaufman et al. in 1964 established that topical 1% cytarabine produces “glittering” corneal epithelial deposits in 92% of rabbit eyes, suppresses tritiated-thymidine incorporation in corneal epithelium and stroma, and disrupts the histochemistry of glycolytic enzymes [41]. Topical deoxycytidine reversed the injury in a dose-dependent manner: 100 µg attenuated and 200 mg completely prevented the deposits.

Balci et al. in 2016 demonstrated, in Wistar rats exposed to intraperitoneal Ara-C 400 mg/kg/day for five days, that NAC 200 mg/kg/day reduced total oxidant status and the oxidative stress index in cornea and conjunctiva by more than 90% [15]. The study is small (*n* = 10 per group) and reports no histology, but the redox signal is consistent with the cerebellar data from the same dose regimen.

Liu et al. in 2024 published the most mechanistically detailed ocular study. In C57BL/6J mice exposed to systemic Ara-C 50 mg/kg/day for seven days, the drug produced corneal epithelial defects, lacrimal-gland hyposecretion, meibomian-gland (MG) plugging with acinar dropout and ductal hyperkeratinization, loss of PPARγ nuclear localization, downregulation of the lipid-metabolic enzymes AWAT2, SOAT1 and ELOVL4, upregulation of HMGCR with cholesterol accumulation and a Keap1-driven collapse of Nrf2/HO-1/SOD1 antioxidant signaling [12]. Oral rosiglitazone (10 mg/kg/day), a PPARγ agonist, normalized corneal fluorescein score, tear volume, lacrimal-gland α-SMA and AQP5, meibomian-gland acinar area, PCNA/P63/Lrig1 proliferation and progenitor markers, K1/K10 hyperkeratinization, the AKT/FoxO1/FoxO3a axis, and oxidative-stress indices.

### 3.6. Hepatotoxicity

Three studies address Ara-C hepatotoxicity, and they converge on a common toxic phenotype, central-lobular and periportal hepatocyte injury with vacuolar degeneration, mononuclear inflammatory infiltrates, portal fibrosis and bile-duct hyperplasia and three different protective agents. Al-Ja mmas et al., in 2020, showed that oral vitamin E at an oral dose of 800 IU daily, given five hours before each intraperitoneal Ara-C dose (50 mg/kg/day for seven days), largely preserved hepatic architecture in New Zealand white rabbits, restored hepatic-cord arrangement with only mild sinusoidal distension and occasional hepatocyte necrosis and some degree of Kupffer cell hyperplasia [33]. Kolure et al., in 2023, in pregnant Sprague-Dawley rats exposed to oral Ara-C 25 mg/kg/day from gestation day 8 to 20, showed that swertiamarin (100 and 200 mg/kg) reversed dose-dependently the rises in MDA, AST, ALT, urea and creatinine and the falls in catalase, SOD, glutathione and glutathione peroxidase, and preserved normal hepatic histology [31]. Dudina et al., in 2018, using an intravenous high-dose regimen (Ara-C 2 g/m^2^ for 5 consecutive days) more representative of clinical exposure, showed that the magnesium 2-aminoethanesulfonate compound LBK-527 (100 mg/kg orally one hour before each Ara-C dose) attenuated the rise in AST, ALT, GGTP and ALP, normalized TNF-α and IL-10, maintained hepatocyte growth factor and restored Bcl-2 and Ki-67 proliferation indices [34].

### 3.7. Bone Marrow and Hematopoiesis

Four studies address what is, paradoxically, both the principal therapeutic target of cytarabine and one of its most dose-limiting toxicities. Zhu et al. in 2013 [27] offered the most mechanistically informative contribution. The PPARγ antagonist BADGE (60 mg/kg intraperitoneally daily for four weeks) reduced Ara-C-driven marrow adipocyte hyperplasia in long-bone and tail-vertebra bone marrow of C57BL/6J mice, accelerated WBC and neutrophil recovery, increased CFU counts at weeks 2–3 post-Ara-C, expanded Ki-67-positive LSK hematopoietic stem and progenitor cells, and remodeled the sinusoidal vasculature [27]. The serum G-CSF level was unchanged, ruling out a G-CSF-mediated mechanism. The conceptual elegance of the result is that PPARγ activation in the marrow stroma drives adipogenesis at the expense of hematopoiesis, and that pharmacological PPARγ blockade therefore reprograms a fatty marrow into a hematopoietic one.

Lee et al., in 2018, tested the CXCR4 antagonist plerixafor in a syngeneic AML model. Single-dose Ara-C 100 mg/kg combined with plerixafor restored bone-marrow sinusoidal vessel density and megakaryocyte counts to levels significantly above either agent alone [19]. Wang et al., in 2018, showed that lienal peptide (1.5 and 4.5 mg/kg/day intraperitoneally) restored splenic CD4^+^, CD8^+^ T-cell and CD19^+^ B-cell frequencies, NK-cell cytotoxicity and macrophage phagocytosis in mice immunosuppressed by Ara-C 250 mg/kg/day for three days [43]. Castañeda-Yslas et al., in 2024, showed that a defined silver-nanoparticle formulation (AgNPs, Argovit-M) administered orally after Ara-C reduced micronucleated polychromatic erythrocyte counts 3.7-fold and micronucleated erythrocyte counts twofold, with the optimal effect achieved when three AgNPs doses followed three Ara-C doses [21].

### 3.8. Chemotherapy-Induced Alopecia

Five studies address Ara-C-induced alopecia in the neonatal rat model. Hussein, in 1995, established the foundational observation that minoxidil, when applied subcutaneously (but not topically as 2% Rogaine in the vehicle used), preserved hair in all 13 pups exposed to Ara-C 75 mg/kg/day for five days [39]. Jimenez et al., in two papers, showed first that recombinant human IL-1β at 0.25 µg daily fully prevented alopecia in Ara-C-treated rats and improved long-term survival from 0% to 100% in a chloroleukaemia co-model [16], and second that the combination of ImuVert and NAC, delivered either subcutaneously or topically as a liposomal suspension, was required to overcome the combined alopecia of cyclophosphamide plus Ara-C, with NAC alone being insufficient [17]. Hagiwara et al., in 2011, showed that topical sodium zinc dihydrolipoylhistidinate (DHLHZn), an α-lipoic acid derivative antioxidant cream, dose-dependently protected against Ara-C alopecia in 8-day-old rats and reduced inflammatory infiltrates and mitochondrial swelling in hair-follicle cells on electron microscopy [42]. Sun et al., in 2009, using a related neonatal rat protocol, showed that AHCC (active hexose correlated compound) given orally at 500 mg/kg/day produced the greatest protection (mild alopecia in 4 of 9 pups vs. uniform severe loss with Ara-C alone) and mitigated chemotherapy-associated weight loss [37].

### 3.9. Reproductive and Developmental Toxicity

Four studies address the reproductive and developmental toxicity of in utero and neonatal Ara-C exposure. Namoju et al. in 2021 demonstrated dose-dependent maternal toxicity, increased fetal resorption and mortality, growth retardation, external malformations, including phocomelia and digit anomalies, delayed ossification, and reduced bone calcium and phosphorus content in pregnant rats exposed to Ara-C at 12.5 or 25 mg/kg/day on gestation days 8–14 [13]. Maternal α-lipoic acid (200 mg/kg orally) co-administered throughout the dosing window normalized every parameter examined. Chilaka et al. in 2024 extended the same paradigm to male reproductive development. F1 male offspring of dams exposed to Ara-C through gestation day 21 showed delayed puberty, reduced testicular, epididymal, prostatic and seminal-vesicle dimensions, elevated testicular MDA with collapsed SOD, glutathione, glutathione peroxidase and catalase activities, depressed plasma testosterone, FSH and LH, falls in 3β-HSD and 17β-HSD activity, reduced sperm count, motility and viability, and seminiferous-tubule atrophy with germ-cell sloughing [22]. Maternal α-lipoic acid largely restored all parameters.

Two foundational studies, by Chaube et al. in 1968 and Kochhar et al. in 1978, established the stage- and dose-dependent teratogenicity of Ara-C and the protective effect of exogenous deoxycytidine [45,46]. Chaube et al. showed in CF Wistar rats that the agent is non-teratogenic on gestation days 5–9 but produces severe malformations on days 10–12 in a sharp dose window. Co-administration of deoxycytidine at 600 mg/kg within a tight time window (20 min before to 10 min after a 150 mg/kg Ara-C dose) completely prevented all malformations, with protection waning rapidly thereafter; dCMP matched deoxycytidine on a molar basis, while CMP, CDP and TdR were ineffective and in some cases worsened fetal mortality [46]. Kochhar et al., in CD-1 mice, refined the picture by demonstrating a strict proximo-distal sequence of limb defects depending on the day of injection (micromelia on day 10.5, phocomelia on 11.0, hemimelia on 11.5, adactyly on 12.0) and by showing that deoxycytidine co-injection (4–10× Ara-C dose) prevented DNA-synthesis arrest, embryolethality and long-bone defects but, intriguingly, induced high rates of polydactyly in rescued limbs [45].

### 3.10. Other Toxicities

Bilgin et al. in 2020 offered the only included study of Ara-C pulmonary protection. Oral rutin (50 mg/kg) administered one hour before each daily intraperitoneal Ara-C dose (200 mg/kg for 14 days) in male Wistar rats prevented the rise in MDA, TOS, TNF-α and NF-κB and the fall in total glutathione and total antioxidant status, eliminated the high-resolution CT signal of pulmonary edema, and restored alveolar architecture [23]. Al-Jammas et al., in 2024, in a separate rabbit study, showed that oral α-tocopherol 800 IU/day attenuated Ara-C-induced parotid salivary-gland injury, with reduction in acinar and ductal necrosis, inflammatory infiltration and TNF-α and partial restoration of Bcl-2 expression [30].

## 4. Discussion

Although cytarabine has been in clinical use for more than six decades and its toxicity profile is well characterized, the experimental evidence on pharmacological mitigation remains predominantly preclinical and is largely derived from single-laboratory, single-agent studies. The present review consolidates this dispersed literature and organizes it according to two questions: which biological mechanisms the reported protective agents share, and which of these mechanisms are closest to clinical translation.

### 4.1. Underlying Pathophysiological Mechanisms in Mitigation Strategies

**Figure 2:**
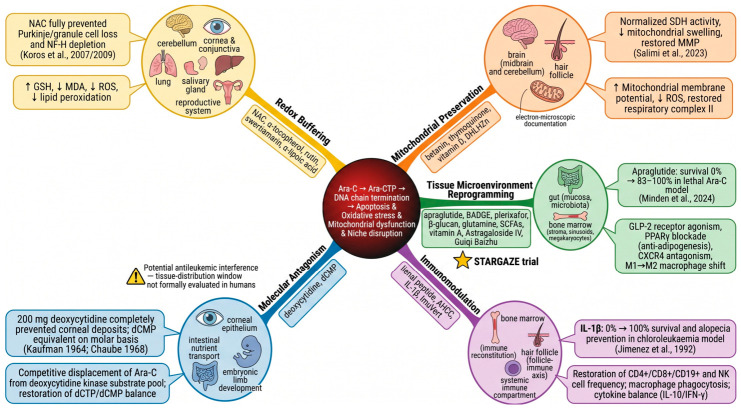
Hub-and-spoke diagram of cytoprotective strategies against Cytarabine (Ara-C)-induced toxicity. The central node represents the primary injury cascade initiated by Ara-C, encompassing DNA chain termination via Ara-CTP incorporation, induction of apoptosis, oxidative stress, mitochondrial dysfunction, and disruption of tissue niches. Five mechanistic categories of cytoprotective intervention radiate from this node: Redox Buffering (NAC, α-tocopherol, rutin; protecting cerebellum, cornea, liver, lung, and reproductive organs), Mitochondrial Preservation (betanin, thymoquinone, vitamin D; protecting brain and hair follicle), Tissue Microenvironment Reprogramming (apraglutide, plerixafor, β-glucan; protecting gut mucosa and bone marrow stroma, with clinical translation highlighted by the STARGAZE trial), Molecular Antagonism (deoxycytidine, dCMP; protecting corneal epithelium and intestinal transport, with a caution flag for potential antileukemic interference), and Immunomodulation (IL-1β, AHCC, lienal peptide; restoring bone marrow immune reconstitution and the follicle-immune axis). ↓, decreased; ↑, increased [7,16,28,29,32,41,46].

Despite the heterogeneity of the protective agents identified, five mechanistic pathways recur across them. Their recurrence in organ systems as distinct as the brain, eye, gut, and bone marrow is unlikely to be coincidental and instead reflects the shared downstream consequences of antimetabolite-driven DNA damage. Such damage simultaneously imposes oxidative stress, disrupts mitochondrial bioenergetics, destabilizes tissue microenvironments, and activates apoptotic and inflammatory signaling in tissues attempting repair. The five pathways described below should therefore be regarded as complementary rather than competing explanations (Figure 2).

Redox buffering was the most frequently represented mechanistic theme. NAC, α-tocopherol, swertiamarin, rutin, and α-lipoic acid share the capacity to replenish reduced glutathione, scavenge reactive oxygen species, and limit lipid and protein oxidation in tissues exposed to Ara-C. The consistency of this redox signal across organ systems as diverse as the cerebellum, cornea, lung, liver, salivary gland, and reproductive tract is notable and is consistent with the biology of an antimetabolite that, beyond its direct DNA chain-termination effect, generates substantial collateral oxidative stress in tissues in which mitotic arrest elicits an abortive repair response. Among the agents identified, NAC was supported by the most internally consistent evidence base. It prevented Ara-C-induced cerebellar ataxia, Purkinje-cell and granule-cell loss, and selective NF-H depletion in two independent Wistar-rat studies using an identical dose regimen, protected corneal and conjunctival oxidative indices at the same dose in the same species, and has generated positive signals in human clinical trials in the hematological oncology setting [47].

Mitochondrial preservation constituted a mechanistically distinct second category. Betanin, thymoquinone, vitamin D, and DHLHZn were reported to act on mitochondrial membrane potential, respiratory-complex II activity, reactive-oxygen-species generation, and mitochondrial swelling rather than on cytosolic redox alone. The electron-microscopic documentation of mitochondrial ultrastructural changes in Ara-C-exposed hair-follicle cells, together with the recovery achieved by DHLHZn, supports the interpretation that integumentary and cerebellar injuries from Ara-C share a substantial mitochondrial component. These observations indicate that the mitochondrion may represent a common substrate underlying otherwise disparate organ injuries associated with this drug class.

Tissue-microenvironment reprogramming represented a distinct and potentially clinically relevant mechanistic category. Apraglutide, BADGE, plerixafor, β-glucan, glutamine, vitamin A, and short-chain fatty acids did not act primarily on parenchymal-cell redox but instead modified the tissue niche, including the gut microbiota, marrow stromal lineage commitment, the hematopoietic-endothelial sinusoidal network, the M1/M2 macrophage balance, and the enterocyte-trophic signaling axis mediated by the GLP-2 receptor. This category yielded the most advanced translational signals identified in the present review: apraglutide is in clinical development [48], plerixafor is already approved for hematopoietic stem-cell mobilization [49], and the principle of microbiota-protective interventions in chemotherapy is established [50]. These findings suggest that further progress may depend less on the identification of additional antioxidants than on the characterization of the niche-level signaling networks that, once disrupted by Ara-C, contribute to the observed organ toxicity.

Molecular antagonism by deoxycytidine and its monophosphate constituted a fourth, historically established mechanism. This principle dates to the 1960s and underlies one of the most reproducible findings in this literature. Deoxycytidine was reported to protect embryonic limb development against Ara-C teratogenesis within a narrow temporal window, to protect the corneal epithelium from topical Ara-C deposits, and to preserve intestinal nutrient transport during systemic Ara-C exposure. A major translational concern is that deoxycytidine, as the endogenous substrate for deoxycytidine kinase (dCK), competes directly with Ara-C for the enzyme responsible for Ara-CTP generation in leukemic blasts, and exogenous deoxycytidine delivery could therefore blunt antileukemic activity as well as rescue normal tissues. The preclinical evidence provides limited but mechanistically interpretable support for the existence of a differential kinetic window. Cytarabine undergoes rapid deamination to inactive uridine arabinoside (Ara-U) by cytidine deaminase (CDA), which is highly expressed in the liver and intestinal epithelium but substantially lower in leukemic blasts; this differential CDA expression is itself a recognized determinant of Ara-C’s hematopoietic selectivity and a pharmacogenomic source of inter-patient toxicity variability [51,52]. The activation of Ara-C to Ara-CTP by dCK in leukemic cells generates an intracellular nucleotide pool with a half-life of several hours to days (due to DNA incorporation), whereas exogenously delivered deoxycytidine is rapidly catabolized by CDA in non-hematopoietic tissues, limiting its duration of competitive inhibition to a narrow window that mirrors the tight temporal protection interval identified experimentally by Chaube et al. (−20 to +10 min relative to Ara-C administration). Whether these differential CDA expression gradients and the differential Ara-CTP retention times between normal and malignant cells are sufficient to permit a pharmacologically exploitable rescue window without proportional antileukemic interference cannot be determined from the available data. Formal evaluation requires contemporary pharmacokinetic studies measuring Ara-CTP concentrations in leukemic blasts and normal intestinal epithelial cells simultaneously under titrated deoxycytidine co-administration in a tumor-bearing model.

The fifth mechanism was immunomodulation. Lienal peptide, AHCC, IL-1β and ImuVert were reported to restore immune-effector populations and cytokine balances suppressed by Ara-C. The clinical context for this category is evolving. Given the increasing integration of immune-based therapy into AML treatment, including venetoclax-based regimens, FLT3 inhibitors, and emerging T-cell engagers, agents that preserve host immunity during Ara-C cytoreduction may have value not only as toxicity mitigators but also as adjuncts to immune-based consolidation strategies.

Table S2 summarizes the evidential strength characterization of lead pharmacological candidates for cytarabine toxicity mitigation.

### 4.2. Translational Insights from Recent Clinical Evidence

The gap between preclinical mitigation signals and clinical application may, in part, reflect structural factors in the incentive landscape of pharmaceutical development. Several recent clinical observations relevant to the major mechanistic pathways identified in this review are summarized below.

#### 4.2.1. GLP-2 Agonism and Intestinal Protection

One of the most notable findings identified in this review was the apraglutide result reported by Minden et al. in 2024 [7]. Administration of this GLP-2 receptor agonist from four days before a cytarabine challenge increased 28-day survival from 0% to 83–100% in a lethal murine mucositis model and was associated with preservation of villous and crypt architecture, maintenance of plasma citrulline as a functional biomarker of enterocyte mass, and normalization of gut microbiota composition, while reported co-administration produced “no impact on leukocyte counts”, confirming the absence of interference with cytarabine-induced myelosuppression. Dose–response analysis indicated near-maximal protection from 1.1 mg/kg, suggesting pharmacological tractability [7].

The mechanistic rationale is well established. GLP-2 is the principal endocrine mediator of enterocyte trophism and crypt-cell anti-apoptosis, and its reduction following chemotherapy-induced injury to intestinal L-cells may exacerbate mucosal breakdown. Endogenous GLP-2 levels increase as a compensatory response to intestinal injury, but this increment appears insufficient under high chemotherapy loading. Long-acting synthetic analogs circumvent the rapid DPP-IV-mediated degradation of native GLP-2 and provide sustained receptor stimulation beyond the period of injury [53,54,55,56].

This signal has also been examined in the clinic in an adjacent indication. The Phase 2 STARGAZE trial (NCT05415410) evaluated apraglutide in 31 patients with steroid-refractory gastrointestinal acute graft-versus-host disease (SR GI aGvHD), a condition in which the intestinal mucosal barrier is severely compromised by both conditioning chemotherapy and allogeneic immune injury. Results reported at EBMT 2025 indicated that apraglutide was well tolerated across high-, low-, and fixed-dosing arms in combination with ruxolitinib. All lower-GI responders at Day 28 maintained their response through Days 56 and 91. The majority of the 31 patients had Grade III–IV aGvHD and Stage 3–4 lower-GI involvement, a population in whom standard salvage therapy is associated with poor outcomes. The safety profile was consistent with the known GLP-2 analog class effect and with the complications expected in the GVHD population [57,58].

Taken together, the murine Ara-C data and the STARGAZE findings are consistent with a common mechanism, whereby GLP-2-receptor agonism protects the intestinal mucosal barrier from chemotherapy-induced apoptotic and inflammatory injury, independent of whether the proximate insult is cytarabine, melphalan, or allogeneic immune activation. A logical next step would be a prospective trial of apraglutide prophylaxis in patients receiving high-dose Ara-C conditioning for allogeneic hematopoietic cell transplantation, with mucositis grade, plasma citrulline, and nutritional outcomes as co-primary endpoints. Separately, a multicenter survey published in 2025 reported favorable results for teduglutide, another GLP-2 analog, in treatment-refractory severe intestinal aGvHD, providing further support for the GLP-2 axis as a therapeutic target in oncology patients whose intestinal barrier has been disrupted by both chemotherapy and immune injury [53,54,55,56,57,58,59].

#### 4.2.2. NAC and Hematopoietic Niche Protection

In the present review, the preclinical signal for NAC was confined to neurotoxicity and ocular toxicity. The agent has, however, entered independent clinical investigation for a related application, namely the improvement of hematopoietic recovery after Ara-C-containing induction chemotherapy in AML, and this clinical evidence reinforces the translational rationale. A series of prospective studies from the Beijing group reported that oral NAC (400 mg three times daily), administered during the peri-transplant and post-chemotherapy period, improved bone marrow endothelial progenitor cell (EPC) function, reduced endothelial ROS concentrations, and accelerated hematopoietic reconstitution in patients receiving cytarabine-containing regimens. A Phase 3 open-label randomized trial (NCT03967665) reported that prophylactic NAC reduced poor graft function and shortened prolonged isolated thrombocytopenia after haploidentical hematopoietic stem cell transplantation, an effect attributed to improved bone marrow endothelial cell dynamics. Of most direct relevance to the present review, a pilot cohort study (NCT06024031) in 30 newly diagnosed AML patients receiving Ara-C-containing induction chemotherapy reported that NAC did not alter complete remission rates (90% vs. 80%, *p* = 0.23) but significantly shortened platelet recovery time among patients achieving remission (19 vs. 22 days, *p* = 0.0001), with higher EPC percentages and enhanced hematopoiesis-supporting EPC function. No significant adverse events were attributable to NAC [47,60,61]. This result is mechanistically interpretable: NAC’s cytoprotective effect in normal tissues operates principally through glutathione replenishment in slowly cycling or post-mitotic cells, whereas the antileukemic activity of Ara-C depends on dCK-mediated phosphorylation to Ara-CTP in rapidly proliferating blasts, a pathway that does not require elevation of cellular redox status for activation. Paradoxically, NAC can induce cytotoxic oxidative stress in human myeloid leukemia cell lines (HL-60 and U937) at pharmacologically achievable concentrations, through myeloperoxidase-dependent conversion of NAC-derived hydrogen peroxide to hypochlorous acid, an effect dependent on the high myeloperoxidase expression that characterizes myeloid lineage cells [62]. Whether this in vitro signal translates to a net proapoptotic effect on blasts in vivo remains uncertain, but the available data at least argue against NAC providing a net survival advantage to AML cells.

#### 4.2.3. Plerixafor and the Hematopoietic Microenvironment

The observation by Lee et al. [19] that plerixafor restored bone marrow sinusoidal vessel density and megakaryocyte counts after Ara-C administration is supported by the subsequent development of plerixafor as an approved hematopoietic stem cell mobilizing agent. Plerixafor is approved by the FDA and EMA for CD34+ cell mobilization in combination with G-CSF in patients with multiple myeloma and non-Hodgkin’s lymphoma undergoing autologous transplantation. CXCR4 signaling maintains hematopoietic stem and progenitor cells (HSPCs) in close apposition to the endosteal niche and regulates sinusoidal integrity through SDF-1α–CXCR4-mediated endothelial survival signaling. Ara-C-induced injury to both HSPCs and the sinusoidal endothelium disrupts this architecture, contributing to prolonged cytopenias that extend well beyond the direct cytotoxic phase. Pharmacological CXCR4 blockade by plerixafor releases HSPCs into peripheral circulation, alters marrow niche stromal lineage commitment, and may accelerate sinusoidal network repair via an as-yet incompletely characterized mechanism. Plerixafor’s regulatory status provides a comprehensive human pharmacokinetic profile. At the approved subcutaneous dose of 0.24 mg/kg, peak plasma concentrations occur within 30–60 min, the elimination half-life is approximately 5.3 h, and the drug is renally excreted with predictable clearance characteristics. This pharmacokinetic profile is fully compatible with co-administration around cytarabine dosing cycles, and dose adjustments for renal impairment are well-established from the autologous transplant setting [63,64].

The Phase I/II PLERIFLAG study, in which 57 patients with primary refractory or early-relapsed AML received plerixafor in combination with a FLAG-Ida regimen incorporating cytarabine at conventional doses, achieved composite CR/CRi rates of 49% at the recommended Phase 2 dose, with allogeneic transplant bridging in 61% of responders and an acceptable induction mortality rate of 7% [65]. Additionally, another Phase 1/2 study reported that plerixafor could be safely combined with cytarabine-containing salvage chemotherapy (MEC regimen) in patients with relapsed or refractory AML, achieving modest leukemic blast mobilization from the marrow niche and producing response rates that were considered encouraging without unacceptable toxicity [66,67]. The rationale in that setting was to sensitize AML blasts to Ara-C by disrupting their stromal protection, an objective that is mechanistically distinct from the host-tissue-protective rationale described by Lee et al. [19] Both lines of investigation, nevertheless, converge on CXCR4-mediated regulation of the microenvironment as a modifiable determinant of response to Ara-C-containing regimens. This dual mechanistic role, comprising AML blast sensitization and rescue of the host hematopoietic niche, identifies plerixafor as a candidate that merits formal evaluation in the context of Ara-C-induced marrow toxicity [63,64,66,67].

#### 4.2.4. Microbiota Modulation

The preclinical observation that short-chain fatty acids and β-glucan modulate the intestinal microbiome and preserve mucosal integrity during Ara-C exposure is consistent with the broader literature on chemotherapy-associated dysbiosis. In a meta-analysis of 12 randomized controlled trials encompassing 1376 cancer patients, multi-strain probiotic preparations were associated with significant reductions in the risk of severe oral mucositis (RR 0.61, 95% CI 0.53–0.72) and all-grade mucositis (RR 0.90, 95% CI 0.82–0.98). Although none of these trials was Ara-C-specific, the underlying principle, namely that microbiota composition is a modifiable determinant of chemotherapy-induced mucosal injury, is directly relevant to the SCFA and β-glucan signals identified in the present review [68].

### 4.3. Limitations

This review has several limitations that should be acknowledged. The included studies are predominantly small, single-center experiments with limited reproducibility and substantial heterogeneity in cytarabine dosing, timing of co-intervention, animal species, and outcome measures. Such variability restricts comparability and precludes meta-analytic synthesis. A limitation of particular weight in this context is the inherent uncertainty of extrapolating rodent pharmacology data to the human clinical setting. Factors of particular relevance to this review include species-specific variation in cytidine deaminase expression (the hepatic enzyme primarily responsible for Ara-C inactivation, which differs substantially between rodent and human tissue), immunological and microbiota differences between germ-free or barrier-maintained laboratory animals and immunocompromised cancer patients, and the absence of the underlying malignancy and its treatment in most included studies. Rodent models of drug toxicity systematically under-represent the complexity of the human cancer chemotherapy setting, and this structural limitation constrains the translational inferences that can be drawn from even well-designed preclinical experiments. As risk-of-bias assessment falls outside the remit of scoping review methodology, the internal validity of individual studies remains uncertain, and the possibility of systematic bias cannot be excluded. Publication bias is likely considerable, as negative or neutral findings are rarely reported in preclinical toxicology. The translational relevance of many interventions is further limited by supra-physiological dosing, non-clinical routes of administration, or experimental conditions that do not fully recapitulate human chemotherapy exposure. Restriction to English-language publications may have resulted in the omission of relevant studies. Finally, the complete absence of cardiotoxicity-mitigation data highlights a critical gap in the current evidence base and underscores the need for targeted investigation.

### 4.4. Future Perspectives

Based on the evidence gaps and mechanistic opportunities identified in this review, the following research priorities are proposed. First, cytarabine is a well-documented cause of pericarditis, and its combination with anthracyclines in standard AML induction produces a clinically significant cardiac adverse event burden. Targeted preclinical characterization of cytarabine-induced pericardial and myocardial injury in validated rodent models, followed by systematic screening of candidate agents with cardiovascular safety precedent, is the highest priority action item. Second, every candidate identified in this review should be validated in at least one independently conducted, adequately powered, tumor-bearing syngeneic model before clinical translation is proposed, as the simultaneous assessment of host-tissue protection and leukemia blast sensitivity in the same animal is non-negotiable for any translational claim. Third, for apraglutide, the most logical immediate next step is the design of a prospective Phase 2 trial of prophylaxis in patients receiving high-dose cytarabine conditioning prior to allogeneic hematopoietic cell transplantation. An appropriate design would include WHO mucositis grade, plasma citrulline at pre-specified timepoints, and rates of parenteral nutrition requirement as co-primary endpoints, with Ara-C pharmacokinetics and post-induction remission status as key secondary endpoints to formally exclude antileukemic interference. Fourth, another priority is independent replication of the most compelling single-laboratory findings. The apraglutide result by Minden et al. [7] represents one study from one laboratory in one murine model and cannot support clinical translation without independent replication in a second animal species, ideally one that includes a tumor-bearing cohort to confirm leukemia-sparing activity, and validation of the plasma citrulline biomarker in that second model. Similarly, the BADGE/PPARγ blockade finding by Zhu et al. (2013) [27] has not been independently reproduced in the twelve years since its publication; independent confirmation of marrow adiposity reduction, HSPC expansion, and sinusoidal restoration is a necessary precondition for clinical evaluation. Finally, for NAC, the phase 3 trial data (NCT03967665) showing improved hematopoietic reconstitution without compromise of complete remission rates justify formal exploration of NAC as a standard peri-transplant adjunct in AML, with prospective biomarker sub-studies to confirm endothelial progenitor cell recovery as the mechanism.

## 5. Conclusions

This scoping review provides the first systematic cartography of pharmacological strategies tested in vivo to mitigate cytarabine-induced multi-organ toxicity. Thirty-six studies spanning six decades converge on five overlapping protective pathways, redox buffering, mitochondrial preservation, tissue-microenvironment reprogramming, molecular antagonism, and immunomodulation, that account for the activity of an otherwise heterogeneous collection of agents across eight organ systems. The two most translatable candidates are apraglutide, which rescued 28-day survival from 0% to 83–100% in a lethal murine Ara-C mucositis model through sustained GLP-2 receptor agonism, and N-acetylcysteine, which fully prevented cerebellar and ocular oxidative injury preclinically and has since generated positive signals in Phase 3 clinical data in AML patients without compromising remission rates. Plerixafor, already an approved mobilizing agent, and PPARγ blockade by BADGE represent additional high-priority candidates for bone marrow microenvironment protection, while the historically validated deoxycytidine antagonism principle warrants formal pharmacokinetic evaluation to confirm a tissue-distribution window that spares antileukemic efficacy.

## Figures and Tables

**Table 1:** The Population–Concept–Context framework.

PCC Element	Inclusion Criteria	Exclusion Criteria
Population	In vivo mammalian models administered cytarabine (Ara-C) as the principal cytotoxic agent. Eligible species: rodents (rat, mouse), lagomorphs (rabbit), or larger mammals. Includes adult, neonatal, pregnant (in utero exposure), and F1-offspring cohorts. Both naïve (healthy) animals and tumor-bearing syngeneic models are eligible.	Purely in vitro preparations (cell lines, organoids, tissue explants). Studies using Ara-C solely as an antineoplastic without dedicated host-tissue toxicity endpoints.
Concept	Any pharmacological co-intervention (prophylactic or concomitant) is tested for its capacity to attenuate Ara-C-induced injury in one or more organ systems. At least one quantitative or histopathological outcome measure of organ injury must be reported.	Agents tested exclusively for anti-leukemic synergy without host-tissue protection endpoints. Narrative reviews, case reports and conference abstracts lacking full text were excluded.
Context	Studies published from database inception to 15 July 2025, identified via PubMed/MEDLINE, Scopus, Cochrane Library and Embase, and Web of Science, supplemented by hand-searching of reference lists. Clinical context: Ara-C use in AML induction and consolidation, ALL, high-grade lymphomas, intrathecal prophylaxis, and emerging combinations.	Studies focusing on non-mammalian organisms. Studies with insufficient methodological detail to permit data charting.

**Table 2:** Comparative summary table of included studies.

Author, Year	Ara-C Regimen	Animal Model	Target Toxicity	Mitigation Agent (Dose, Route)	Principal Protective Outcome
Liu R et al., 2024 [12]	Ara-C 50 mg/kg i.p. × 7 d	C57BL/6J mice, M, 6–8 wk	Ocular: meibomian gland dysfunction, lacrimal hyposecretion	Rosiglitazone 10 mg/kg/day p.o. × 7 d (PPARγ agonist)	Normalized corneal fluorescein score, tear volume, MG acinar area; restored PPARγ/AWAT2/SOAT1/ELOVL4 and Nrf2/HO-1/SOD1 antioxidant signaling.
Namoju R et al., 2021 [13]	Ara-C 12.5 or 25 mg/kg i.p./day, GD 8–14	Pregnant rats (180–220 g)	Developmental: resorptions, malformations, ossification defects	α-Lipoic acid 200 mg/kg p.o. daily, GD 8–14	Normalized maternal weight, placental antioxidant indices, fetal survival, malformation rates and bone Ca/P (*p* < 0.05–0.001 vs. Ara-C).
de Souza Silva PM et al., 2018 [14]	Ara-C 1.8 mg/mouse i.p. ×4 q12h × 2 d	BALB/c mice, ~70 d	GI mucositis, leucopenia, DNA damage	β-D-glucan 80 mg/kg + glutamine 150 mg/kg p.o. × 21 d	Fully preserved crypt mitoses; halved leucopenia; reduced comet-assay DNA damage by >50%.
Balci YI et al., 2016 [15]	Ara-C 400 mg/kg i.p. × 5 d	Wistar rats, mature, ~250 g	Ocular: corneal/conjunctival oxidative stress	NAC 200 mg/kg/day i.p. × 5 d	TOS and oxidative stress index reduced > 90% vs. Ara-C (*p* < 0.01).
Jimenez JJ et al., 1992 (IL-1β) [16]	Ara-C 20 mg/kg i.p. × 7 d	7-day-old Fisher rats, C51 chloroleukaemia	Alopecia + leukemia survival	rHu-IL-1β 0.25 µg i.p. × 7 d	Survival 100% (vs. 0% vehicle), 9/10 with no alopecia.
Jimenez JJ et al., 1992 (ImuVert/NAC) [17]	Ara-C 50 mg/kg i.p. × 4–5 d	Sprague-Dawley rats, 7-day-old	Chemo-induced alopecia (CTX+Ara-C)	ImuVert 10 µg + NAC 4 mg s.c. or topical	Liposomal topical ImuVert+NAC: 9/9 thick hair regrowth, 3/9 full protection. NAC alone is insufficient.
Li JJ et al., 2023 [18]	Ara-C 100 mg/kg i.p. × 7 d	C57BL/6 mice, M, 8–10 wk	GI mucositis	Astragaloside IV 10/20/40 mg/kg i.p. × 7 d	40 mg/kg attenuated weight loss & ileal damage; restored ZO-1/occludin; reduced M1 macrophages and PI3K/AKT signaling.
Lee JY et al., 2018 [19]	Ara-C 100 mg/kg i.p. single dose	C57BL/6J mice, 7 wk, C1498 AML	BM microenvironment: sinusoids, megakaryocytes	Plerixafor (CXCR4 antagonist) s.c.	Restored sinusoid density (~37/field) and megakaryocytes (~13/field), significantly above either agent alone (*p* < 0.01).
Ramos MG et al., 1997 [20]	Ara-C 3.6 mg/mouse q12h × 2 or 4 d	Swiss NMRI mice	GI mucositis	Oral SCFAs (acetate:propionate:butyrate)	Markedly reduced villus atrophy, necrosis and inflammation; preserved mucosal protein and nucleotides.
Castañeda-Yslas IY et al., 2024 [21]	Ara-C 6 mg/kg i.p., 1 or 3 doses	BALB/c mice, M, 5–6 wk	Genotoxicity & myelosuppression	AgNPs (Argovit-M) 6 mg/kg p.o. ×3, post-Ara-C	3.7-fold ↓ MNPCE and 2.0-fold ↓ MNE; sequential Group 6 is most effective.
Chilaka KN et al., 2024 [22]	Ara-C 12.5/25 mg/kg i.p./day, GD 8–21	F1 male rat offspring, PND 73	Reproductive: testicular development	Maternal α-lipoic acid 200 mg/kg p.o., GD 8–21	Normalized testis dimensions, testosterone/FSH/LH, sperm metrics, tubule architecture, antioxidant enzymes.
Bilgin AO et al., 2020 [23]	Ara-C 200 mg/kg i.p. × 14 d	Wistar rats, M, 260–280 g	Pulmonary edema & oxidative injury	Rutin 50 mg/kg p.o., 1 h before Ara-C	Normalized MDA, TOS, TNF-α, NF-κB, tGSH, TAS; eliminated CT edema; restored alveolar architecture.
Chu W et al., 2023 [24]	Ara-C 100 mg/kg i.p. × 7 d	C57BL/6 mice, M, 6–8 wk	GI mucositis	GQBZP 11 or 22 g/kg p.o. (3 d before + 10 d)	22 g/kg restored weight, villus/crypt ratio; suppressed M1 macrophages via JAK2/STAT1 inhibition.
Guzmán DC et al., 2018 [25]	Ara-C 0.6 g/kg i.v. × 5 d	Wistar rats, M, ~80 g, 4 wk	Neurotoxicity (dopamine, oxidative stress)	Stevia 0.6 g/kg p.o. × 5 d	Partially restored GSH and lowered TBARS vs. Ara-C; region-specific monoamine modulation.
Guzmán DC et al., 2016 [26]	Ara-C 70 mg/kg i.p. × 5 d	Wistar rats, M, ~100 g	Neurotoxicity (cortical oxidative stress)	Oleic acid 1500 µL/kg i.p. × 5 d	Restored GSH, Na^+^,K^+^-ATPase in cortex; preserved regional dopamine (*p* < 0.001).
Zhu RJ et al., 2013 [27]	Ara-C 0.5 g/kg i.p. × 4 d	C57BL/6J mice, F, 6–8 wk	BM adipogenesis, hematopoietic recovery	BADGE 60 mg/kg i.p. × 4 wk (PPARγ antagonist)	Reduced marrow adipocytes, accelerated WBC/neutrophil recovery, expanded Ki-67^+^ LSK cells, restored sinusoids.
Koros C et al., 2007 [28]	Ara-C 400 mg/kg i.p. × 5 d	Wistar rats, M, ~250–350 g	Cerebellar dysfunction	NAC 200 mg/kg/day p.o. × 14 d (7 d pre + 7 d co)	Fully prevented ataxia, rotarod deficits, Purkinje/granule cell loss, NF/calbindin alterations.
Koros C et al., 2009 [29]	Ara-C 400 mg/kg i.p. × 5 d	Wistar rats, M, ~10 wk	Cerebellar NF-H loss	NAC 200 mg/kg/day p.o. × 14 d	Preserved NF-H to control levels; restored NF immunoreactivity in the molecular layer.
Al-Jammas S et al., 2024 [30]	Ara-C 60 mg/kg i.p. × 10 d	New Zealand white rabbits, ~4 mo	Parotid salivary gland injury	α-Tocopherol 800 IU/day p.o. × 10 d	Preserved gland architecture; reduced TNF-α from grade 3 to 1; restored Bcl-2.
Minden MD et al., 2024 [7]	Ara-C 30 mg/kg i.p. bid × 5 d	BALB/cAnNCRL mice, M, 12–18 wk	GI mucositis and mortality	Apraglutide 0.33–3.3 mg/kg s.c., −4 d to +12	Survival 83–100% vs. 0% Ara-C alone (*p* < 0.0001); preserved villi/crypts; maintained citrulline and microbiota.
Kolure R et al., 2023 [31]	Ara-C 25 mg/kg p.o./day, GD 8–20	Pregnant Sprague-Dawley rats	Maternal hepatotoxicity	Swertiamarin 100 or 200 mg/kg p.o.	Dose-dependent normalization of AST/ALT, urea, creatinine, antioxidant enzymes; preserved hepatic histology.
Salimi A et al., 2023 [32]	Ara-C 70 mg/kg i.p. × 5 d	Wistar rats, M, 200 ± 20 g	Brain mitochondrial dysfunction	Betanin 25 mg/kg, Vit D 500 IU/kg, or thymoquinone 0.5 mg/kg i.p.	Normalized AChE/BChE, redox balance, mitochondrial SDH, swelling, ROS, MMP (*p* < 0.01–0.001 vs. Ara-C).
Al-Jammas S et al., 2020 [33]	Ara-C 50 mg/kg i.p. × 7 d	New Zealand white rabbits, 3 mo	Hepatotoxicity	Vitamin E 800 IU p.o., 5 h before Ara-C	Largely preserved hepatic architecture; restored hepatic cords; eliminated portal fibrosis and vascular congestion.
Dudina MO et al., 2018 [34]	Ara-C 2 g/m^2^ i.v. × 5 d	Wistar rats, M/F, 180–220 g	Acute cytotoxic liver injury	LBK-527 (Mg-taurate) 100 mg/kg p.o. 1 h pre-Ara-C	Normalized AST/ALT/GGTP/ALP; restored IL-10; reduced TNF-α; maintained HGF; ↑ Bcl-2 and Ki-67.
Porsani MYH et al., 2017 [35]	Ara-C 15 mg/kg i.p. q12h × 4 doses	BALB/c mice, ~50 d	GI mucositis and immune suppression	β-glucan 80 mg/kg + glutamine 150 mg/kg p.o. × 21 d	Highest IL-13/IL-10, lowest leucocyte depletion and IFN-γ; preserved villus-crypt morphology.
Elli M et al., 2009 [36]	Ara-C 3.6 mg/mouse i.p. × 5 d	BALB/c mice, M, 8–10 wk	Jejunal mucosal injury	Vitamin A 5000 IU/kg p.o. × 7 d	Preserved villus length and crypt depth; reduced necrosis (*p* < 0.001 vs. Ara-C).
Sun B et al., 2009 [37]	Ara-C 30 mg/kg/day i.p. × 7 d	Sprague-Dawley rat pups, 8 d	Alopecia	AHCC 500 mg/kg/day p.o.	4/9 pups with only mild alopecia (vs uniform severe with Ara-C); also mitigated weight loss.
Ramos MG et al., 1999 [38]	Ara-C 3.6 mg/mouse i.p. × 2 d (days 8–9)	Germ-free mice	GI mucositis (microbiota-independent)	Oral or intragastric SCFA (35/15/9 mM) × 9 d	Greater villus and total mucosal length vs. Ara-C; oral route slightly superior; colon length preserved.
Hussein AM, 1995 [39]	Ara-C 75 mg/kg/day i.p. × 5 d	Sprague-Dawley rat pups, 8 d	Alopecia	Minoxidil s.c. 0.2 mg/pup × 6 d	All 13 pups showed local hair retention over the injection site.
Guzmán DC et al., 2024 [40]	Ara-C 0.08 mM i.p. single dose	Wistar rats, F, ~70 g, 4 wk	Neurochemical oxidative stress	Oligoelement mix (Fe/Zn/Mn/Se/Cr/Cu/Mo/I/F) 50 µL i.p.	↓ TBARS in cortex, striatum, cerebellum; modulated regional dopamine; restored Na^+^, K^+^-ATPase.
Kaufman HE et al., 1964 [41]	1% topical Ara-C drops q2h × 5 d	New Zealand white rabbits	Corneal epithelial toxicity	Topical deoxycytidine (100 µg–200 mg)	200 mg dose completely prevented epithelial deposits and normalized DNA and enzyme histochemistry.
Hagiwara S et al., 2011 [42]	Ara-C 20 mg/kg/day i.p. × 7 d	Wistar rat pups, 8 d	Alopecia	Topical DHLHZn 0.5% or 1% cream × 12 d	1% DHLHZn: alopecia score ~control (*p* < 0.05); reduced follicle inflammation and mitochondrial swelling.
Wang J et al., 2018 [43]	Ara-C 250 mg/kg/day i.p. × 3 d	C57BL/6 mice, M, 18–20 g	Immunosuppression	Lienal peptide 1.5 or 4.5 mg/kg/day i.p.	Restored splenic CD3/CD4/CD8 and BM CD19 cells; NK cytotoxicity; macrophage phagocytosis.
Chen T, 1982 [44]	Ara-C 50 mg/kg/day i.p. × 5 d	Swiss-Webster mice, M, 30–40 g	Intestinal nutrient transport	2′-Deoxycytidine 100 mg/kg i.p.	Restored 3-O-methyl-glucose transport to control; prevented weight loss.
Kochhar DM et al., 1978 [45]	Ara-C 2–200 mg/kg i.p. single dose, GD 10.5–12	ICR (CD-1) mice	Embryonic limb development	2′-Deoxycytidine 4–10× Ara-C dose	Prevented DNA-synthesis arrest, cell death and long-bone defects; induced polydactyly in rescued limbs.
Chaube S et al., 1968 [46]	Ara-C 2.5–900 mg/kg i.p. single dose, GD 5–12	CF Wistar rats	Teratogenesis (cleft palate, encephalocele, limb defects)	Deoxycytidine 600 mg/kg or dCMP	Completely prevented all malformations within a −20 to +10 min window; dCMP equivalent on a molar basis.

Abbreviations: Ara-C, cytarabine (cytosine arabinoside); AML, acute myeloid leukemia; NAC, N-acetylcysteine; AHCC, active hexose correlated compound; AgNPs, silver nanoparticles; BADGE, bisphenol A diglycidyl ether; DHLHZn, sodium zinc dihydrolipoylhistidinate; dCMP, deoxycytidine monophosphate; SCFA(s), short-chain fatty acids; GQBZP, Guiqi Baizhu prescription; GLP-2, glucagon-like peptide-2; PPARγ, peroxisome proliferator-activated receptor gamma; CXCR4, C-X-C chemokine receptor type 4; G-CSF, granulocyte colony-stimulating factor; LSK, lineage^−^ Sca-1^+^ c-Kit^+^ hematopoietic stem and progenitor cells; CFU, colony-forming unit; WBC, white blood cell count; MDA, malondialdehyde; TBARS, thiobarbituric acid reactive substances; TOS, total oxidant status; TAS, total antioxidant status; tGSH, total glutathione; GSH, reduced glutathione; SOD, superoxide dismutase; ROS, reactive oxygen species; MMP, mitochondrial membrane potential; SDH, succinate dehydrogenase; NF-κB, nuclear factor kappa-light-chain-enhancer of activated B cells; TNF-α, tumor necrosis factor-alpha; IL-1β, interleukin-1 beta; IL-6, interleukin-6; IL-10, interleukin-10; IFN-γ, interferon-gamma; iNOS, inducible nitric oxide synthase; JAK2/STAT1, Janus kinase 2/signal transducer and activator of transcription 1; PI3K/AKT, phosphoinositide 3-kinase/protein kinase B; Nrf2, nuclear factor erythroid 2-related factor 2; HO-1, heme oxygenase-1; Keap1, Kelch-like ECH-associated protein 1; Bcl-2, B-cell lymphoma protein 2; Ki-67, proliferation antigen KI-67; PCNA, proliferating cell nuclear antigen; α-SMA, alpha smooth muscle actin; AQP5, aquaporin-5; NF-H/NF-M/NF-L, neurofilament heavy/medium/light subunit; AChE, acetylcholinesterase; BChE, butyrylcholinesterase; HGF, hepatocyte growth factor; AST, aspartate aminotransferase; ALT, alanine aminotransferase; ALP, alkaline phosphatase; GGTP, gamma-glutamyl transpeptidase; FSH, follicle-stimulating hormone; LH, luteinizing hormone; 3β-HSD, 3-beta-hydroxysteroid dehydrogenase; 17β-HSD, 17-beta-hydroxysteroid dehydrogenase; MNPCE, micronucleated polychromatic erythrocytes; MNE, micronucleated erythrocytes; MG, meibomian gland; AWAT2, acyl-CoA wax alcohol acyltransferase 2; SOAT1, sterol O-acyltransferase 1; ELOVL4, elongation of very long chain fatty acids protein 4; HMGCR, 3-hydroxy-3-methylglutaryl-CoA reductase; ZO-1, zonula occludens-1; rHu-IL-1β, recombinant human interleukin-1 beta; NK, natural killer cell; CD4^+^/CD8^+^/CD19^+^, T-cell and B-cell surface markers; EPC, endothelial progenitor cell; DPP-IV, dipeptidyl peptidase IV; i.p., intraperitoneal; i.v., intravenous; s.c., subcutaneous; p.o., per os (oral); bid, twice daily; q12h, every 12 h; GD, gestation day; PND, postnatal day; F, female; M, male; F1, first filial generation; wk, weeks; mo, months; ↓, decreased; ↑, increased.

## Data Availability

No new data were created or analyzed in this study. Data sharing is not applicable to this article.

## Supplementary Materials

The following supporting information can be downloaded at: https://www.mdpi.com/article/10.3390/cancers18132060/s1, Table S1: PRISMA-ScR checklist, Table S2: Evidential strength characterization of lead pharmacological candidates for cytarabine toxicity mitigation.
