# Supplementary material for: Pharmacological Strategies for Mitigating Cytarabine-Induced Multi-Organ Toxicity: A Scoping Review on Mechanisms, Efficacy and Clinical Implications
Source: Cancers (Basel). 2026 Jun 25;18(13):2060. doi: 10.3390/cancers18132060 (PMC13359649; doi:10.3390/cancers18132060)
Supplement: Supplementary file 1 [file cancers-18-02060-s001.zip › cancers-4380704-Table S2.pdf]

Table S2. Evidential strength characterization of lead pharmacological candidates for cytarabine toxicity mitigation.

Agent / Category	Studies Identified	Independent Replication	Tumur-Model Data	Leukemia-Sparing Evidence	Clinical Evidence	Translational Readiness
Plerixafor	1 (BM microenvironment)	No	Yes: syngeneic AML model [33]	Equivocal: dual role; normal HSPC mobilization and AML blast chemosensitisation [64, 65, 66]	FDA/EMA approved; combined with cytarabine in PLERIFLAG trial [66]	**Very High** (for host niche protection)
Apraglutide	1 (GI mucositis)	No	No (naïve BALB/c mice)	Yes: no impact on leukocyte counts in murine model [7]; GLP-2 receptor absent from leukemic blasts	Phase 2 STARGAZE trial (SR GI aGvHD) [58]	**High**
N-acetylcysteine (NAC)	3 (neurotox, ocular)	Yes: two independent Wistar-rat studies, identical regimen, same group (Koros 2007, 2009)	No (healthy animals)	Yes: Phase 3 RCT (NCT03967665) and AML pilot (NCT06024031) showed no CR rate impairment [60-62]; NAC cytotoxic to myeloid lines in vitro [63]	Phase 3 RCT, Phase 1/2 pilot; both encouraging results	**High**
Short-chain fatty acids	2 (GI mucositis)	Yes: same group, two distinct murine models (conventional and germ-free)	No	Not tested	None	Moderate

α-Lipoic acid	2 (developmental tox.)	Yes: Namoju 2021 & Chilaka 2024, independent groups, overlapping endpoints	No	Not tested	None	Low–Moderate
Deoxycytidine / dCMP	3 (ocular, GI, teratogenesis)	Partial: three independent groups, similar directional findings	No	Unknown: direct dCK competition raises theoretical antileukemic interference risk	None	Low (requires formal PK evaluation)
BADGE (PPARγ antagonism)	1 (BM adipogenesis)	No	No	Not tested; PPARγ role in AML biology incompletely characterised	None	Low
Betanin / thymoquinone / vitamin D	1 combined study (neurotox)	No	No	Not tested; thymoquinone and vitamin D have reported anti-leukaemic properties	None	Low

BM, bone marrow; CR, complete remission; dCK, deoxycytidine kinase; GI, gastrointestinal; HSPC, haematopoietic stem and progenitor cell; PK, pharmacokinetics; SR, steroid-refractory; aGvHD, acute graft-versus-host disease.
